# SARS-CoV-2 Vaccine Effectiveness against Omicron Variant in Infection-Naive Population, Australia, 2022

**DOI:** 10.3201/eid2906.230130

**Published:** 2023-06

**Authors:** Lauren E. Bloomfield, Sera Ngeh, Gemma Cadby, Kate Hutcheon, Paul V. Effler

**Affiliations:** The University of Notre Dame Australia, Fremantle, Western Australia, Australia (L.E. Bloomfield);; Western Australia Department of Health, East Perth, Western Australia, Australia (L.E. Bloomfield, S. Ngeh, G. Cadby, K. Hutcheon, P.V. Effler)

**Keywords:** COVID-19, 2019 novel coronavirus disease, coronavirus disease, severe acute respiratory syndrome coronavirus 2, SARS-CoV-2, viruses, respiratory infections, zoonoses, vaccine effectiveness, Omicron, SARS-CoV-2–naive, Australia

## Abstract

SARS-CoV-2 transmission in Western Australia, Australia, was negligible until a wave of Omicron variant infections emerged in February 2022, when >90% of adults had been vaccinated. This unique pandemic experience enabled assessment of SARS-CoV-2 vaccine effectiveness (VE) without potential interference from background immunity from prior infection. We matched 188,950 persons who had a positive PCR test result during February–May 2022 to negative controls by age, week of test, and other possible confounders. Overall, 3-dose VE was 42.0% against infection and 81.7% against hospitalization or death. A primary series of 2 viral-vectored vaccines followed by an mRNA booster provided significantly longer protection against infection >60 days after vaccination than a 3-dose series of mRNA vaccine. In a population free from non–vaccine-derived background immunity, vaccines against the ancestral spike protein were ≈80% effective for preventing serious outcomes from infection with the SARS-CoV-2 Omicron variant.

Until February 2022, Western Australia (WA), Australia, successfully delayed sustained transmission of SARS-CoV-2 by using rigorous international quarantine and state border entry restrictions, supported by comprehensive local outbreak control measures when breaches occurred. Data corroborating no substantive community transmission of SARS-CoV-2 in WA until February 2022 include the incidence of reported laboratory-confirmed SARS-CoV-2 infections (Figure 1) and serologic testing of blood donors. By the time WA lifted restrictions and experienced the first wave of SARS-CoV-2 infection, >90% of the state’s residents >16 years of age had received >2 doses of a SARS-CoV-2 vaccine (*1*). The WA public health laboratory, PathWest Laboratory, determined which SARS-CoV-2 variants were circulating in the state during the first pandemic wave. During the study period (February 1–May 31, 2022), all 2,695 specimens for which lineage could be assigned were the Omicron variant, of which 2,668 (99%) were designated as BA.1 or BA.2 sublineage (Figure 2).

**Figure 1:**
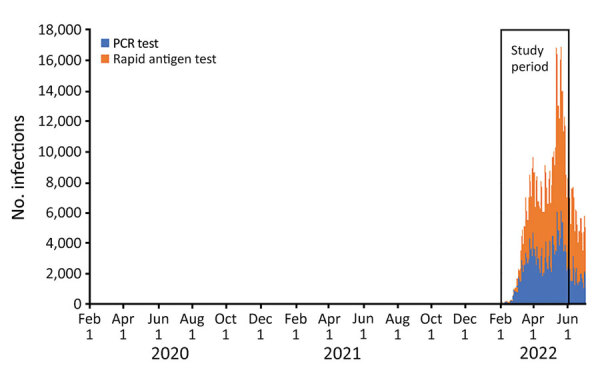
Number of SARS-CoV-2–positive test results reported by day, by test type, Western Australia, Australia, February 1–June 30, 2022. Source: COVID-19 Public Health Operations, WA Health (D. Barth, COVID-19 Public Health Operations, WA, Australia, pers. comm., email, 2022 Dec 1).

**Figure 2:**
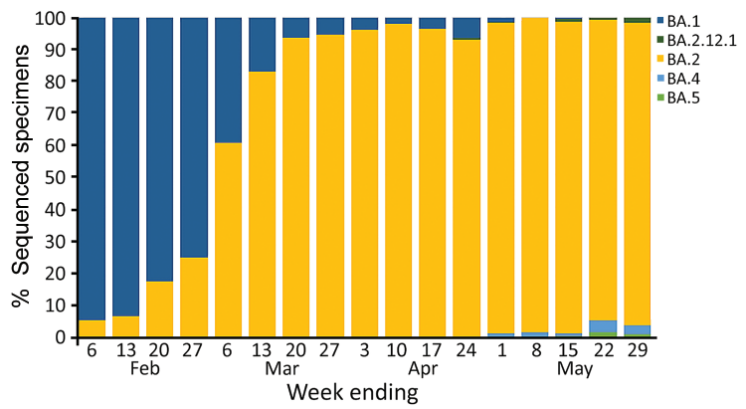
Weekly proportion of assigned lineages for sequenced SARS-CoV-2 specimens, by week of sample collection, Western Australia, Australia, February 1–May 31, 2022. Source: PathWest Laboratory Medicine (C. Sikazwe, Pathwest Laboratory, WA, Australia, pers. comm., email, 2022 July 15).

This unique pandemic experience enabled real-world assessment of vaccine effectiveness (VE) without potential bias caused by population-level background immunity resulting from prior infection. With this study, we assessed VE for mRNA vaccines and viral-vectored SARS-CoV-2 vaccines against laboratory-confirmed infection and severe illness caused exclusively by the Omicron variant in a SARS-CoV-2–naive population. This work was approved by the WA Department of Health Human Research Ethics Committee (RGS0000005522).

## Methods

### Study Design

We used a test-negative case–control design to compare persons >16 years of age who had positive PCR SARS-CoV-2 test results with matched controls who were SARS-CoV-2 negative. We compared the odds of vaccination among those with PCR-confirmed SARS-CoV-2 infection or severe COVID-19 with the odds of vaccination among matched PCR-confirmed negative controls.

### Data Linkage

In early 2020, WA created a statewide linked data repository to collect information essential for public health management of the pandemic, including SARS-CoV-2 vaccinations that began in February 2021 (*2*). In brief, COVID-19 vaccination data from the Australian Immunisation Register (AIR; Appendix) were linked to existing WA Department of Health data collections, including statewide hospital admission data, mortality data, and SARS-CoV-2 pathology test results.

### Ascertainment of Infection

All persons with >1 SARS-CoV-2 PCR tests performed within the study period were eligible for inclusion. We included in the analysis only a person’s first positive or negative PCR test result during the study period. Persons who initially tested negative but subsequently tested positive during the study period were removed from the negative test pool and reclassified as a case-patient (i.e., each person appeared once in the dataset, as either a case-patient or a control). Because the reason for SARS-CoV-2 testing was not recorded in the data repository, we excluded persons with >20 test results reported during January 2020–May 2022 because those persons were probably subjected to regular asymptomatic screening for employment purposes. We also excluded persons with only SARS-CoV-2 rapid antigen test (RAT) results because negative RAT results were not required to be reported and compliance with mandatory reporting of positive RAT results is unknown.

### Vaccination Data

For this analysis, we established vaccination status at the time of the person’s eligible SARS-CoV-2 PCR test result by linking the person to their respective SARS-CoV-2 immunization history in AIR. We included in our analysis persons who received any dose of vaccine >14 days before the eligible PCR result and excluded persons who had received a vaccine dose <14 days before the eligible PCR result. We defined unvaccinated persons as those who had no record of receiving a SARS-CoV-2 vaccine dose before their linked PCR test result.

The vaccines analyzed were the adenovirus-vectored vaccine produced by AstraZeneca (ChAdOx1, https://www.astrazeneca.com) and the mRNA vaccines produced by Pfizer-BioNTech (BNT162b2, https://www.pfizer.com) and Moderna (mRNA-1273, https://www.modernatx.com). We defined a homologous 3-dose vaccination schedule as 2 doses of an mRNA vaccine and an mRNA booster dose, regardless of brand/manufacturer, and a heterologous 3-dose vaccination schedule as 2 doses of ChAdOx1 followed by an mRNA booster dose, regardless of brand/manufacturer. Matched pairs in which the case-patient or control had received a protein-based SARS-CoV-2 vaccine, a ChAdOx1 booster dose, or a mixed 2-dose primary schedule (ChAdOx1 and an mRNA vaccine) were few; we excluded those pairs from subanalysis examining heterologous and homologous vaccine combinations.

### Hospitalization and Mortality Data

During the study period, we extracted data from the WA Hospital Morbidity Data Collection (*3*) and linked them to all SARS-CoV-2 test results for specimens collected. We defined a SARS-CoV-2 hospitalization as >1 inpatient admissions 0–7 days after the date of a positive PCR result. To reduce potential bias that might be introduced through routine preadmission SARS-CoV-2 screening, we excluded admissions for patients indicated by the specialty of the admitting clinician to be unlikely to have been hospitalized for treatment of COVID-19 and for patients admitted for boarding purposes (Table 1). We also performed supplementary sensitivity analyses, which included all hospital admissions regardless of admitting clinician specialty or those defined as a COVID-19 hospitalization on the basis of select codes from the International Statistical Classification of Diseases and Related Health Problems, Tenth Revision, Australian Modification (ICD-10-AM).

**Table 1:** Clinician specialties for which hospital admissions were excluded from the analysis of SARS-CoV-2 vaccine effectiveness, Western Australia, Australia, February 1, 2022–May 31, 2022

Specialty
Child psychiatry/psychology
In vitro fertilization
Gynecology oncologist
Oncology
Psychogeriatrics
Psychiatry
Radiology
Nephrology/dialysis
Obstetrics
Burns
Dental surgery
Gynecology
Ophthalmology
Oral surgery
Orthopedics
Plastic and reconstructive surgery
Renal transplant surgery
Spinal surgery
Radiation oncology
General practitioner obstetrics

The WA Registry of Births, Deaths and Marriages (https://www.wa.gov.au), the mandatory repository for death reports in WA, provided mortality data. We defined SARS-CoV-2–associated deaths as death from any cause 0–30 days after an eligible PCR test and included those cases in the analysis. We defined severe disease as SARS-CoV-2 hospitalization, an associated death, or both.

To identify pre-existing medical conditions that could potentially increase a person’s risk for SARS-CoV-2 infection or severe disease, we reviewed any hospital admissions in the 24 months before their eligible PCR test. We selected 14 diagnostic categories and the ICD-10-AM codes used to define them, based on previous literature (Table 2) (*4*). If a person had any of the ICD-10-AM codes listed as either a principal or secondary diagnosis for an admission in the 24 months before their eligible PCR test, they were assigned a score of 1 for the corresponding diagnostic category. We then summed the total number of unique diagnostic categories assigned a score of 1 for each person, yielding a score of 0–14 (0 if there had been no admissions or only admissions that did not include any of the selected ICD-10-AM codes). We used this aggregate comorbidity score as a proxy index of underlying conditions and controlled for it in the analyses.

**Table 2:** ICD-10-AM hospital admission codes used to identify and categorize underlying conditions and construct a comorbidity score for each person included in a study of SARS-CoV-2 vaccine effectiveness, Western Australia, Australia, 2022*

Underlying condition category	ICD-10-AM codes
Respiratory disease	J40–J47; J81; J84; E84
Diabetes	E10–E14
Anemia/splenic issues	D50–D59; D60–D63; D73
Down syndrome	Q90
Cancer	C00–C97 excl C44
Kidney disease	N18–N19; N00–N16; N25–N29
Immunocompromise	D80–D89; B24
Dementia	F00–F03; G30
Cardiac disease incl hypertension and arrhythmia	I20–I28; I05–I10; I47–I50
Stroke/TIA	G45; H34; I60–I69
Liver disease	K71–K77
Obesity	E66
Rheumatoid arthritis	M05–M06
Ulcerative colitis/Crohn’s disease	K50–K51

*ICD-10-AM, International Statistical Classification of Diseases and Related Health Problems, Tenth Revision, Australian Modification; TIA, transient ischemic attack.

### Statistical Analyses

We assessed VE by using conditional logistic regression of matched case–control pairs. We calculated adjusted odds ratios (aORs) by using the clogistic function in the Epi package in R version 4.1.0 (The R Foundation for Statistical Computing, https://www.r-project.org). Vaccine effectiveness was defined as (1 – aOR) × 100 and is presented with 95% CIs. We assessed differences between odds ratios by calculating the absolute difference between the log odds and the SEs of the difference. We calculated p values by using z-scores; to account for multiple testing, we considered p<0.01 significant.

Case-patients and controls were matched on week of eligible PCR test, age group (16–19 years, then 10-year intervals thereafter), sex, Aboriginality (defined as Aboriginal and/or Torres Strait Islander ancestry), Index of Relative Socioeconomic Advantage and Disadvantage decile (*5*) (a score of 1 demonstrating relatively greater disadvantage and a score of 10 indicating a relative lack of disadvantage), and comorbidity score using by the MatchIt package (*6*) on a 1:1 basis. We randomly sorted all eligible case-patients and controls by using the sample function before matching.

We calculated effectiveness by comparing unvaccinated persons with those who had received either 2 or 3 doses of vaccine. We also performed subanalyses to examine VE for breakthrough infection by time since last vaccine dose and between the homologous and heterologous 3-dose vaccination schedule. Analysis of VE against severe disease included positive case-patients (by PCR) with severe disease who, along with their matched controls, received 2 or 3 vaccine doses.

## Results

**Figure 3:**
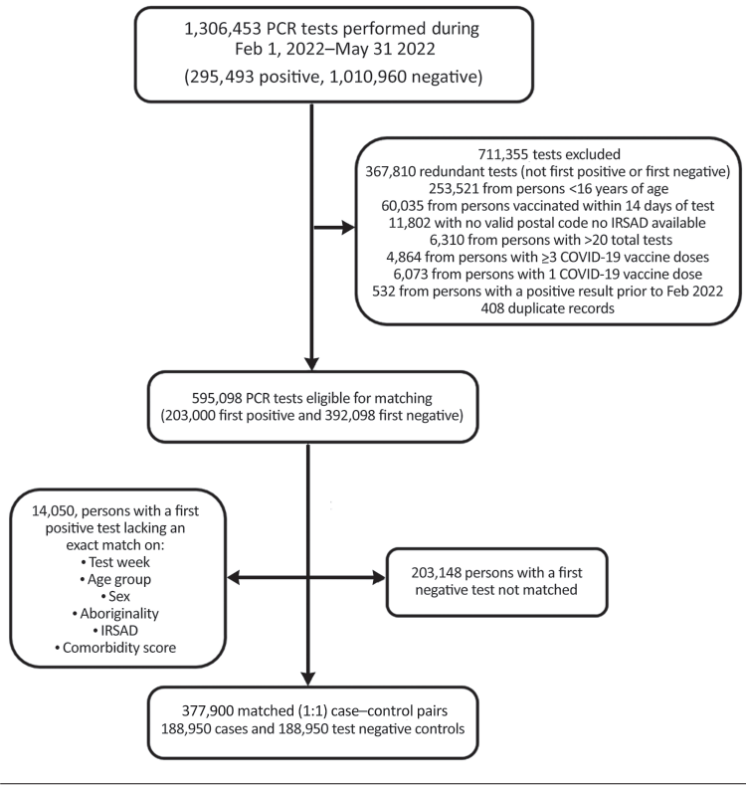
Selection of SARS-CoV-2 cases positive by PCR and of negative controls for analysis of vaccine effectiveness, Western Australia, Australia, February 1–May 31, 2022. IRSAD, Index of Relative Socioeconomic Advantage and Disadvantage.

A total of 1,306,453 PCR tests were reported for specimens collected during February 1, 2022–May 31, 2022, from which 188,950 positive case-patients and 188,950 negative matched controls were eligible for inclusion in the analysis (Table 3; Figure 3). Of the 377,900 study participants, 30,420 (8%) were unvaccinated at the time of testing, 84,237 (22%) had received 2 doses, and 263,243 (70%) had received 3 doses.

**Table 3:** Demographic characteristics of matched samples for analysis of SARS-CoV-2 vaccine effectiveness, Western Australia, Australia, February 1–May 31, 2022*

Characteristic	Unvaccinated, no. (%), n = 30,420	Two doses, no. (%), n = 84,237	Three doses, no. (%), n = 263,243	Total, no. (%), n = 377,900
Age group, y				
16–19	1,470 (4.8)	13,645 (16.2)	14,451 (5.5)	29,566 (7.8)
20–29	9,851 (32.4)	28,569 (33.9)	50,886 (19.3)	89,306 (23.6)
30–39	9,083 (29.9)	20,395 (24.2)	58,914 (22.4)	88,392 (23.4)
40–49	4,294 (14.1)	11,672 (13.9)	58,712 (22.3)	74,678 (19.8)
50–59	2,519 (8.3)	5,709 (6.8)	42,446 (16.1)	50,674 (13.4)
60–69	1,646 (5.4)	2,517 (3.0)	21,903 (8.3)	26,066 (6.9)
70–79	849 (2.8)	932 (1.1)	9,577 (3.6)	11,358 (3.0)
>80	708 (2.3)	798 (0.9)	6,354 (2.4)	7,860 (2.1)
Sex				
F	14,180 (46.6)	40,940 (48.6)	144,180 (54.8)	199,300 (52.7)
M	15,972 (52.5)	43,143 (51.2)	118,699 (45.1)	177,814 (47.1)
Unspecified	268 (0.9)	154 (0.2)	364 (0.1)	786 (0.2)
Aboriginal Status				
Non-Aboriginal	29,367 (96.5)	80,418 (95.5)	259,701 (98.7)	369,486 (97.8)
Aboriginal	1,053 (3.5)	3,819 (4.5)	3,542 (1.3)	8,414 (2.2)
No. comorbidities				
0	29,269 (96.2)	80,583 (95.7)	246,896 (93.8)	356,748 (94.4)
1	221 (0.7)	852 (1.0)	3,929 (1.5)	5,002 (1.3)
2	596 (2.0)	2,046 (2.4)	9,008 (3.4)	11,650 (3.1)
3	46 (0.2)	120 (0.1)	778 (0.3)	944 (0.2)
4	195 (0.6)	404 (0.5)	1,815 (0.7)	2,414 (0.6)
>5	93 (0.3)	229 (0.3)	817 (0.3)	1,142 (0.3)
IRSAD				
1	345 (1.1)	390 (0.5)	515 (0.2)	1,250 (0.3)
2	2,090 (6.9)	5,893 (7.0)	12,425 (4.7)	20,408 (5.4)
3	658 (2.2)	2,242 (2.7)	5,636 (2.1)	8,536 (2.3)
4	2,893 (9.5)	9,375 (11.1)	24,962 (9.5)	37,230 (9.9)
5	2,649 (8.7)	9,784 (11.6)	26,867 (10.2)	39,300 (10.4)
6	3,446 (11.3)	9,734 (11.6)	25,666 (9.8)	38,846 (10.3)
7	3,296 (10.8)	10,479 (12.4)	28,123 (10.7)	41,898 (11.1)
8	3,967 (13.0)	13,371 (15.9)	39,974 (15.2)	57,312 (15.2)
9	6,186 (20.3)	13,719 (16.3)	51,589 (19.6)	71,494 (18.9)
10	4,890 (16.1)	9,250 (11.0)	47,486 (18.0)	61,626 (16.3)

*IRSAD, Index of Relative Socioeconomic Advantage and Disadvantage.

### VE against Breakthrough Infection of Any Severity

**Figure 4:**
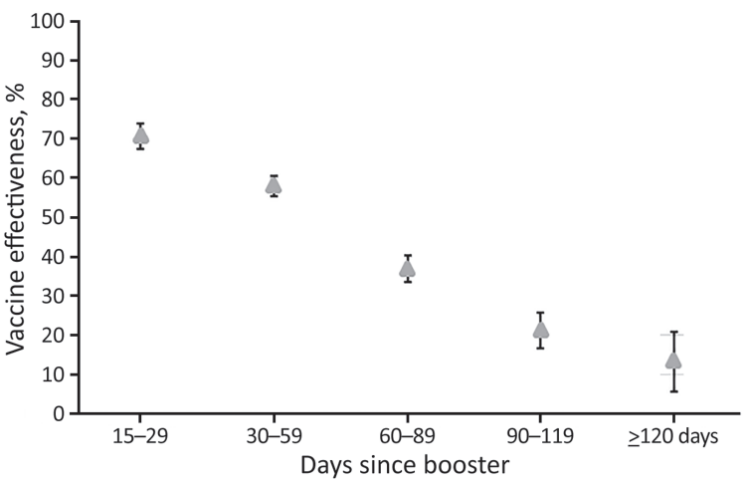
Vaccine effectiveness against breakthrough infection of any severity, by time since first booster dose versus unvaccinated controls, Western Australia, Australia, February 1–May 31, 2022. Error bars indicate 95% CIs.

Overall, in adjusted analyses comparing those who were unvaccinated with those who had received 2 vaccine doses, VE for preventing PCR-confirmed infection of any severity was 24.9% (95% CI 21.2%–28.4%), increasing to 42.0% (95% CI 40.2%–43.6%) for persons who had received 3 doses (Table 4). Breakthrough infection of any severity waned notably after a booster dose. For those who had received a booster dose 15–29 days before testing, VE was 70.7% (95% CI 67.4%–73.7%) but fell to 13.5% (95% CI 5.6%–20.8%) for those whose booster was administered >120 days before testing (Figure 4). The median time between the most recent vaccine dose and PCR test date was 21 days longer for persons who received 2 doses (101 days) than for those who received 3 doses (80 days); given the waning immunity after 3 doses, it is possible that increased time since vaccination contributed to lower VE estimates for 2 versus 3 doses.

**Table 4:** Vaccine effectiveness against breakthrough SARS-CoV-2 infection of any severity, 2 or 3 doses versus unvaccinated, Western Australia, Australia, February 1, 2022–May 31, 2022*

Case-patients			Controls		Vaccine effectiveness, % (95% CI)†
Vaccinated	Unvaccinated		Vaccinated	Unvaccinated	
16,306	6,060		17,292	5,074	24.9 (21.2–28.4)
103,741	14,299		108,863	9,177	42.0 (40.2–43.6)

*Case-patient (SARS-CoV-2 positive) and control (SARS-CoV-2 negative) pairs (infection status determined by PCR) matched by age group, sex, Aboriginality, testing week, Index of Relative Socioeconomic Advantage and Disadvantage decile and comorbidities. †Vaccine effectiveness = 1 − adjusted odds ratio (ascertained by using conditional logistic regression of matched pairs).

### VE against Severe Disease

Hospitalizations and deaths in the study cohort were rare. Among the 188,950 matched case-patients, there were 264 deaths within 30 days of testing and 1,456 hospital admissions within 7 days, excluding persons admitted under clinician specialties (Table 1).

Overall, VE against severe disease for 2 doses of vaccine was 41.9% (95% CI 4.8%–64.5%) and increased to 81.7% (95% CI 73.9%–87.2%) for 3 doses (Table 5). The number of case-patients with severe disease in individual time strata was insufficient to permit meaningful VE estimates to be generated by time since last vaccination.

**Table 5:** Vaccine effectiveness against severe COVID-19, 2 or 3 doses versus unvaccinated, Western Australia, Australia, February 1, 2022–May 31, 2022*

Vaccination status	Case-patients, hospital admission or death			Controls		Vaccine effectiveness, % (95% CI)†
	Vaccinated	Unvaccinated		Vaccinated	Unvaccinated	
Two doses	131	69		148	52	41.9 (4.8–64.5)
Three doses	795	259		982	72	81.7 (73.9–87.2)

*Severe disease was defined as hospitalization for SARS-CoV-2 infection with 7 d of positive SARS-CoV-2 PCR results and/or death from any cause. †Vaccine effectiveness calculated with conditional logistic regression by using case–control pairs matched by week of testing, age group, sex, Aboriginality, Index of Relative Socioeconomic Advantage and Disadvantage, and comorbidity score.

### VE for Homologous versus Heterologous 3-Dose Series

Our analysis of VE by different vaccine series was restricted to 100,142 case-patients who received either a homologous or heterologous series of 3-dose vaccination (Table 6), as defined previously, and their matched controls. Persons who were vaccinated according to the heterologous versus homologous schedule were older (median age at test 65 vs. 38 years), more likely to be male, and more likely to have >1 comorbidity (Table 7).

**Table 6:** Dosing schedule for case-patients and controls included in the heterologous versus homologous 3-dose vaccination schedule analysis of SARS-CoV-2 vaccine effectiveness, Western Australia, Australia, February 1, 2022–May 31, 2022*

Dose 1	Dose 2	Dose 3	No. (%)
Homologous 3-dose vaccination schedules			
BNT162b2 mRNA	BNT162b2 mRNA	BNT162b2 mRNA	112,939 (75.7)
BNT162b2 mRNA	BNT162b2 mRNA	mRNA-1273	30,676 (20.6)
mRNA-1273	mRNA-1273	mRNA-1273	3,721 (2.5)
mRNA-1273	mRNA-1273	BNT162b2 mRNA	1,775 (1.2)
mRNA-1273	BNT162b2 mRNA	BNT162b2 mRNA	22 (0)
mRNA-1273	BNT162b2 mRNA	mRNA-1273	9 (0)
BNT162b2 mRNA	mRNA-1273	mRNA-1273	7 (0)
BNT162b2 mRNA	mRNA-1273	BNT162b2 mRNA	2 (0)
Heterologous 3-dose vaccination schedules			
ChAdOx1	ChAdOx1	BNT162b2 mRNA	18,925 (78.2)
ChAdOx1	ChAdOx1	mRNA-1273	5,261 (21.8)

*BNT162b2, Pfizer-BioNTech (https://www.pfizer.com); ChAdOx1, AstraZeneca (https://www.astrazeneca.com); mRNA-1273, Moderna (https://www.modernatx.com).

**Table 7:** Demographic characteristics of matched pairs for analysis for homologous versus heterologous 3-dose schedule SARS-CoV-2 vaccine effectiveness subanalysis, Western Australia, Australia, February 1, 2022–May 31, 2022*

Characteristic	Homologous schedule, no. (%)	Heterologous schedule, no. (%)
Age group, y		
16–19	8,409 (5.4)	0 (0.2)
20–29	33,199 (23.5)	249 (6.8)
30–39	42,223 (29.0)	268 (6.0)
40–49	44,699 (28.4)	291 (2.7)
50–59	17,565 (11.3)	4,449 (16.8)
60–69	1,442 (1.2)	10,897 (38.4)
70–79	370 (0.3)	5,923 (21.0)
>80	1,244 (0.9)	2,109 (8.0)
Sex		
F	84,961 (55.8)	11,458 (46.2)
M	63,950 (43.9)	12,706 (53.4)
Unspecified	240 (0.3)	25 (0.5)
Aboriginal Status		
Non-Aboriginal	147,475 (98.7)	24,069 (99.2)
Aboriginal	1,676 (1.3)	117 (0.8)
No. comorbidities		
0	143,910 (96.6)	19,940 (85.0)
1	1,656 (1.0)	780 (2.7)
2	2,997 (1.9)	2,284 (8.0)
3	153 (0.1)	258 (0.9)
4	337 (0.2)	624 (2.3)
>5	98 (0.1)	300 (1.1)
IRSAD		
1	306 (0.3)	31 (0.5)
2	6,641 (4.7)	1,024 (4.7)
3	3,074 (2.1)	561 (2.3)
4	12,710 (8.6)	2,649 (10.7)
5	14,401 (9.4)	2,756 (10.5)
6	14,992 (10.2)	1,846 (8.4)
7	15,669 (10.5)	2,268 (9.5)
8	23,381 (15.4)	2,825 (11.7)
9	30,236 (20.4)	4,827 (20.4)
10	27,741 (18.5)	5,399 (21.4)

*IRSAD, Index of Relative Socioeconomic Advantage and Disadvantage.

**Figure 5:**
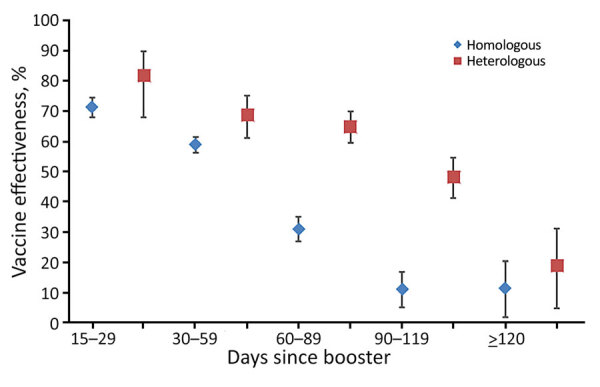
Vaccine effectiveness against breakthrough infection of any severity, by time since booster vaccination, for homologous (all mRNA vaccines) versus heterologous (ChAdOx1 primary, mRNA booster) vaccination series, Western Australia, Australia, February 1–May 31, 2022. Error bars indicate 95% CIs.

VE was higher, although not significantly, up to 60 days after administration of the booster dose among persons who received the homologous or heterologous 3-dose vaccination series. However, VE was significantly higher among those who received a heterologous 3-dose vaccination series 60 days through <120 days of follow-up (Figure 5).

Restricting the samples to those <60 years of age to account for differences in behavior that may contribute to differential breakthrough infection rates yielded similar results. We observed the same pattern of waning immunity in the homologous and the heterologous groups, and protection was significantly higher from 60 to <120 days of follow-up (data not shown) for those on the heterologous schedule

Consistent with the results for breakthrough infection, VE against severe disease was ≈10% higher for those on the heterologous schedule (85.7%, 95% CI 73.1%–92.4%) than on the homologous mRNA schedule (75.8%, 95% CI 61.5%–84.8%), although that difference was not statistically significant in the context of a relatively small number of severe outcomes (p = 0.19). Low numbers of severe outcomes also precluded further analysis of effectiveness by time since vaccination.

### Sensitivity Analyses

We performed sensitivity analyses to explore the potential effects of measurement bias, including the effect of using different options for defining disease severity and selecting covariates for matching case-patients to controls. We found no statistically significant effect on VE for severe disease when we restricted hospitalizations to those likely to be for COVID-19 by selecting specific ICD-10-AM codes used for the primary diagnosis instead of the specialty of the admitting clinician (point estimates for VE after 3 doses changed from 79.2% when restricted by primary diagnosis codes to 81.7% when restricted by admitting clinician specialty). In addition, we explored using different time frames to capture hospitalizations after a positive PCR, specifically 0–14, 2–14, and 2–9 days. VE estimates for severe disease did not differ significantly when we varied the window for hospitalization from 0–7 days to these alternate time frames.

Last, we re-ran the analyses while individually and sequentially adding covariates of interest to the matching algorithm to explore potential effects of confounding. Matching by age group and week of PCR test was sufficient to produce stable VE estimates that were statistically similar to those observed when matching on the full set of covariates for infection of any severity and severe disease.

## Discussion

The ability of WA to limit the introduction of SARS-CoV-2 and prevent sustained local transmission 2 years into the pandemic provides a rare opportunity to assess VE in a population without potential confounding from prior asymptomatic or undiagnosed infection, factors that could affect VE assessments performed in almost all other settings. An assessment of VE in Sydney, New South Wales, Australia, also cited low rates of SARS-CoV-2 background infections in its study population, but WA and metropolitan Sydney have had different pandemic experiences (*4*). Cohort follow-up for the New South Wales study began in January 2022, by which time 157,880 SARS-CoV-2 infections had been reported in the Sydney metropolitan area, largely the result of a protracted outbreak of the Delta variant in 2021 (*7*). Given that an estimated 40% of all SARS-CoV-2 infections are thought to be asymptomatic (*8*) and that the degree of underascertainment of those with mild illness who do not seek testing is unknown, it is difficult to quantify with certainty the extent of prior SARS-CoV-2 infection in the Sydney cohort. In contrast, the very low number of locally acquired infections identified before mid-February 2022 in WA (in the context of high rates of testing and robust contact tracing) and the extremely low rate of nucleocapsid antibody positivity among WA blood donor specimens collected during late February–early March 2022 (*9*) provide convincing evidence that prior SARS-CoV-2 infection was close to negligible among the population used for our analysis.

Most of our key findings are consistent with those from previous studies (*10*–*12*). First, overall VE against breakthrough infection of any severity across the full study period for persons who received 2 doses of vaccine was low (24.9% vs. 42.0% for those who received 3 doses). Subanalyses demonstrated that for those who received a booster dose, VE against any infection was near 71% at 15–29 days after vaccination but declined to <14% by 120 days**.**

Second, protection against severe disease after 3 vaccine doses was much higher than that after 2 doses; overall VE was estimated to be >80%. This finding is relevant because there has been concern that the level of protection afforded by vaccines based on the spike protein of the ancestral strain would be inadequate against Omicron variants, necessitating development of new bivalent vaccines designed to enhance the immune response to Omicron-specific epitopes (*10*).

This study showed considerable protection from monovalent vaccines against clinically severe illness during an exclusively Omicron wave among a population with negligible background immunity from exposure to the ancestral lineage or previous variants of concern. Our study definitively demonstrates that ancestral strain vaccines still provide substantial protection against severe disease caused by newer variants among a population free from potential confounding of previous immunity conferred by natural infection with an earlier variant.

Vaccine-derived cross-protection observed in our setting may have implications for SARS-CoV-2 vaccine science going forward, specifically for assessing the need to continually design new variant-specific vaccines as the virus evolves. Although greater follow-up time is needed, these data support the hypothesis that vaccine effectiveness against severe disease caused by Omicron is likely to be substantially higher than the estimates against symptomatic disease, as has been observed for previous variants of concern (*13*–*15*).

We observed that a heterologous schedule consisting of 2 primary doses of ChAdOx1 followed by a booster dose of an mRNA vaccine provided significantly greater protection against infection >60 days after the last dose, compared with a homologous 3-dose series of mRNA-based vaccines (mostly 3 doses of BNT162b2). This finding is unlikely to be explained by recipients of the heterologous vaccine schedule being inherently less prone to COVID-19 because after the association between ChAdOx1 and thrombotic events was identified, ChAdOx1 was almost entirely administered to persons >60 years of age across Australia. However, in accordance with guidance in Australia about brand-based differences for recommended first- and second-dose intervals, the median time between 2 doses of an mRNA primary series in our cohort was 27 days; for a ChAdOx1 primary series, it was 84 days. It is therefore possible that the longer interval between doses contributed to the more durable protection we observed with the heterologous schedule.

A study of Omicron infections in England found that VE against symptomatic illness was similar between those who received 2 doses of either ChAdOx1 or BNT162b2 followed by a booster dose of BNT162b2, specifically 62.4% and 67.2% given 2–4 weeks after the booster, falling to 39.6% and 45.7%, respectively, after >10 weeks (*10*). One key difference between our setting and that of the study in England is that in late 2020, the United Kingdom began recommending up to 12 weeks between the first and second doses of BNT162b2, a practice uncommon in the WA cohort, and a longer interval between BNT162b2 doses has been shown to enhance immunogenicity (*13*).

Alternatively, the superior performance of the heterologous schedule in protecting against Omicron infection in WA may be a real phenomenon, unmasked without interference from substantial levels of background immunity caused by prior infections with earlier variants. The enhanced immune response, and in some instances clinical protection against non-Omicron variants, produced by heterologous vaccination schedules has been documented in a variety of settings (*14*,*15*).

Among the limitations of our study, our analysis was restricted to using PCR test results from licensed laboratories to determine a person’s status as a case-patient or control and excluded self-administered RATs reported by the general public. This approach was necessary because RAT results were not systematically linked to other datasets used in this analysis. However, even if possible, including RAT results in our setting would have been methodically undesirable because reporting of negative RAT results is not mandatory and the degree of underreporting of positive RAT results by the public is not quantifiable. Furthermore, because there may be relevant differences between those who seek PCR testing and those who choose to self-administer a RAT, including only positive RAT results would have the potential to introduce significant bias when using a VE study design that explicitly relies on matching test-positive case-patients to similar test-negative controls.

Another limitation is that although we attempted to control for relevant confounders, we were limited by the information available in the data repository, and the potential for residual confounding remains. For example, without knowing the reasons for obtaining a PCR test, we could not exclude tests performed for asymptomatic screening, perhaps before an elective hospital admission or for work requirements. Instead, to account for this limitation, we created a surrogate by excluding all tests for persons with >20 tests over the study period and admissions to hospital services not likely to be treating COVID-19. Likewise, because we were not able to directly access information about a person’s comorbidities, we created a comorbidity score based on hospital ICD-10-AM codes accumulated in the previous 2 years. Although we believe those strategies reduced confounding, they are probably imperfect proxies.

Among the strengths of this study, first, the entire cohort of persons >16 years of age who were tested for SARS-CoV-2 by PCR was eligible for inclusion, which resulted in a large sample, probably representative of the population at risk during the study period. Second, we accessed all SARS-CoV-2 PCR results from every private and public clinical laboratory in the state notified to the WA Department of Health as required by law. Third, we used a comprehensive linked data collection, which included hospitalization data from public and private hospitals and mortality data for the entire state. Fourth, vaccination status was obtained from a population-based, whole-of-life, mandatory national immunization register. AIR data are generally considered to be of good quality (*16*), and there was a strong incentive for persons to be sure that their COVID-19 vaccination record was accurate and up to date because activities such as employment and attending public venues were dependent on vaccination status (*17*). Last, the pandemic experience in WA enabled assessment of VE in a SARS-CoV-2 infection–naive cohort, which eliminated potential interference from non–vaccine-derived prior immunity.

In summary, as a result of a sustained, concerted effort to prevent introduction and spread of SARS-CoV-2 during the first 2 years of the pandemic, WA was in a unique position to evaluate, on the basis of the ancestral spike protein, the effectiveness of vaccines to prevent infection and severe disease caused by the Omicron variant in a population that had not experienced prior local SARS-CoV-2 transmission. We demonstrated that VE against infection of any severity was ≈70% up to 1 month after vaccination but waned to very low levels by 4 months. Compared with a homologous 3-dose mRNA vaccine series, a heterologous series consisting of 2 doses of ChAdOx1 followed by an mRNA booster provided significantly longer protection after 60 days, up to 4 months. Protection against hospitalization and death after 3 doses was high (i.e., ≈80%), reinforcing the value of SARS-CoV-2 vaccines for preventing serious outcomes from SARS-CoV-2 infection.

Appendix Background information for study of SARS-CoV-2 vaccine effectiveness against Omicron variant in infection-naive population, Australia, 2022.
