# Supplementary material for: SARS-CoV-2 Vaccine Effectiveness against Omicron Variant in Infection-Naive Population, Australia, 2022
Source: Emerg Infect Dis. 2023 Jun;29(6):1162–72. doi: 10.3201/eid2906.230130 (PMC10202853; doi:10.3201/eid2906.230130)
Supplement: Appendix — Background information for study of SARS-CoV-2 vaccine effectiveness against Omicron variant in infection-naive population, Australia, 2022. [file 23-0130-Techapp-s1.pdf]

Article DOI: https://doi.org/10.3201/eid2906.230130

EID cannot ensure accessibility for supplementary materials supplied by authors. Readers who have difficulty accessing supplementary content should contact the authors for assistance.

SARS-CoV-2 Vaccine Effectiveness against Omicron Variant in Infection-Naive Population, Australia, 2022

Appendix

Until October 14, 2022, SARS-CoV-2 testing was notifiable in WA and all clinical pathology laboratories were required to report results to the WA Department of Health. During January 1, 2020–January 31, 2022, a total of 2,003,941 SARS-CoV-2 PCR test results were reported; 1,527 persons, <0.001% of the state's 2.8 million population) had ≥1 more positive result (*1*). All persons with a positive result were investigated by contact tracers, and 134 (8%) of all reported infections were attributed to transmission within WA.

The low incidence of SARS-CoV-2 infections reported before February 2022 is corroborated by serologic testing of a representative sample of WA blood donor specimens collected between February 23 and March 3, 2022, when just 5 of 999 (0.5%; 95% CI 0.2%–1.2%) specimens were positive for nucleocapsid antibodies, indicative of prior infection (*2*). In contrast, 983 (98.4%; 95% CI 97.4%–99.1%) of the specimens were positive for spike antibodies, reflecting high vaccination rates, consistent with data from the Australian Immunisation Register (AIR, https://www.servicesaustralia.gov.au/australian-immunisation-register). Reporting COVID-19 vaccinations to this national population-based register is mandatory. As of February 1, 2022, AIR data indicated that 91% (1,928,167) of the population ≥16 years of age in WA had received 2 doses of a COVID-19 vaccine and 38% (780,374) of the eligible population ≥18 years of age had received a third (booster) dose (*3*,*4*).

References

1. Government of Western Australia Department of Health. COVID-19 update 31 January 2022 [cited 2023 Jan 30]. https://ww2.health.wa.gov.au/Media-releases/2022/COVID-19-update-31-January-2022

2. Australian COVID-19 Serosurveillance Network. Seroprevalence of SARS-CoV-2-specific antibodies among Australian blood donors: Round 2 update [cited 2023 Jan 30]. https://kirby.unsw.edu.au/sites/default/files/kirby/report/COVID19-Blood-Donor-Report-May-Jun-2022_0.pdf

3. Australian Government. COVID-19 vaccine roll-out: 01 February 2022 [cited 2023 Jan 30]. https://www.health.gov.au/sites/default/files/documents/2022/02/covid-19-vaccine-rollout-update-1-february-2022.pdf

4. Australian Bureau of Statistics. National, state and territory population. 2022 [cited 2023 Jan 30]. https://www.abs.gov.au/statistics/people/population/national-state-and-territory-population/jun-2020
